# IQ score gains over 65 years worldwide: Cross-temporal meta-analysis datasets

**DOI:** 10.1016/j.dib.2019.104884

**Published:** 2019-11-26

**Authors:** Peera Wongupparaj, Veena Kumari, Robin G. Morris

**Affiliations:** a Cognitive Science and Innovation Research Unit (CSIRU), College of Research Methodology and Cognitive Science, Burapha University, Thailand; b Department of Psychology, King's College Institute of Psychiatry, Psychology, and Neuroscience, London, UK; c Centre for Cognitive Neuroscience, College of Health and Life Sciences, Brunel University London, UK

**Keywords:** Flynn effect, Fluid intelligence, A cross-temporal meta-analysis, Colour progressive matrices, Standard progressive matrices, Advanced progressive matrices

## Abstract

The observed gain in IQ scores over time has been examined and supported. Nonetheless, this phenomenon (also called Flynn effect) may depend on age groups and country types. This article provides raw data from three standardized intelligence tests, namely, Coloured Progressive Matrices (CPM), Standard Progressive Matrices (SPM), and Advanced Progressive Matrices (APM). The datasets contain mean IQ scores from APM, CPM, and SPM, and standard deviations, sample sizes, years of publication, participants' groups, types of countries, country-based samples, and gender of participants. This data was obtained from 199, 369, and 176 individual study samples for CPM, SPM, and APM, respectively, and covered a period of 65 years (1950–2014). There were 202,468 participants in total. An analysis and interpretation of results based on a cross-temporal meta-analysis for mean IQ scores from CPM, SPM, and APM over time can be found in the article “A Cross-Temporal Meta-Analysis of Raven's Progressive Matrices: Age groups and developing versus developed countries” (Wongupparaj, Kumari, Morris, 2015) [1]. These datasets can provide an extensive overview of the literature on Flynn effect across age groups, countries, and gender. In addition, they can serve as a useful starting point for further meta-analyses of IQ scores derived from CPM, SPM, and APM.

**Table:** Specifications Table

Subject	Psychology
Specific subject area	Applied Psychology; Experimental and Cognitive Psychology; Psychology (General)
Type of data	Sav files (SPSS files)
How data were acquired	Raw data were collected from the research articles published in English, obtained using scientific literature search methodology.
Data format	Raw
Parameters for data collection	Each study was included in a final dataset for the Cross-Temporal Meta-Analysis (CTMA) research if it could provide the mean and/or standard deviation IQ scores for the Coloured Progressive Matrices (CPM), Standard Progressive matrices (SPM), and Advanced Progressive Matrices (APM). Additional information collected were the authors, mean age of participants, countries of participants, sample sizes, careers, genders reported in each study, incorporated into the final datasets for CTMA research.
Description of data collection	All studies were identified through three main databases, that is, PubMed, ScienceDirect, and SpringerLink. All relevant studies were reviewed and assessed according to inclusion and exclusion criteria (see ‘Experimental designs, Materials, and Methods’). The target parameters were then extracted from each included study.
Data source location	The CPM, SPM, and APM datasets include respectively 38, 50, and countries worldwide.
Data accessibility	Repository name: Mendeley data Data identification number: https://doi.org/10.17632/9jkchm9tnw.4 Datasets can be accessed at Mendeley data: Wongupparaj, Peera (2019), “Datasets for Coloured, Standard, and Advanced Progressive Matrices (CPM, SPM, and APM) over 65 years (1950–2014)”, Mendeley Data, V4, https://doi.org/10.17632/9jkchm9tnw.3 The direct URL to data: https://doi.org/10.17632/9jkchm9tnw.3
Related research article	**Author's name**: Wongupparaj, P., Kumari, V., & Morris, R. G. **Title**: A Cross-Temporal Meta-Analysis of Raven's Progressive Matrices: Age groups and developing versus developed countries **Journal**: Intelligence **DOI**: https://doi.org/10.1016/j.intell.2014.11.008

**Table:** 

**Value of the Data** • The data offers an extensive overview of the parameters for all three versions of Raven's Progressive Matrices (RPM) (i.e. CPM, SPM, and APM) across age groups, countries, gender, occupations, sample sizes, and years of publication. • The datasets will be important for modelling and testing the psychometric properties (i.e. reliability and validity) of the RPM over time and contexts. • The obtained data of these datasets can be used in further studies to investigate the IQ rise or decline (i.e. the Flynn effect vs the Anti-Flynn effect) across age groups, countries, gender, and occupations. • The datasets will be useful to provide several strategies to estimate and reduce the Flynn effect on the interpretation of the IQ tests. • The datasets will be of useful to provide a starting point for conducting further meta-analysis or computing an updated meta-analytic estimate from 1950 to present across three versions of the RPM.

## 1 Data

The data for CPM, SPM, and APM are in SPSS files (sav). The CPM file contains 10 variables from 199 independent samples, namely, authors of each study, mean age of participants (2.90–80 years), years of publication (1956–2014) countries of participants (38 countries), type of countries (73% developed and 27% developing countries), sample sizes (6–2255 participants), careers (83.9% students), gender in each study (6.7% male only, 1.0% female only, and 92.3% mixed).

The SPM file also contains 10 variables from 369 independent samples: Authors of each study, mean age of participants (5.50–79 years), years of publication (1950–2014), countries of participants (50 countries), type of countries (73% developed and 27% developing countries), sample sizes (6–2255 participants), careers (83.9% students), gender in each study (6.7% male only, 1.0% female only, and 92.3% mixed).

Finally, the APM file offers 10 variables from 176 independent samples, that is, authors of each study, mean age of participants (8.68–46.05 years), years of publication (1967–2014), countries of participants (28 countries), type of countries (94% developed and 6% developing countries), sample sizes (8–7335 participants), careers (73% students), gender in each study (87% mixed, 10.5% male only, and 2.8% female only).

The complete references for included studies are additionally available in the Appendix A: Supplementary data section of Ref. [1].

## 2 Experimental design, materials, and methods

### 2.1 Data and literature search strategy

The key terms used were “Raven's Progressive Matrices”, “RPM”, “Coloured Progressive Matrices”, “CPM”, “Standard Progressive Matrices”, “SPM”, “Advanced Progressive Matrices”, and “APM”, and these used search strings were primarily located through only data bases, PubMed, ScienceDirect, and SpringerLink. PubMed contains references and abstracts, with extensive studies in life sciences and biomedical topics. ScienceDirect covers scientific and medical research, including main studies in physical sciences and engineering, life sciences, health sciences and social sciences and humanities. Finally, SpringerLink is a record of scientific, technological, and medical studies. The search strategy was for studies published between 1936 and 2014.

### 2.2 Inclusion and exclusion criteria

Studies were contained in the datasets were reviewed according to the inclusion and exclusion criteria, namely the studies had to report the mean and/or standard deviation IQ scores of standard versions of the IQ test The target parameters from studies with a test-retest method were also collected but only pre-test scores were recorded and if several studies investigated the same dataset and/or sample, the mean and/or standard deviation IQ scores were treated as a single data point. In addition, the target parameters from clinical studies were included if the given parameters were reported for healthy controls. The exclusion criteria included the mean and/or standard deviation IQ scores from short-form, odd-or even-item, and modified versions of the CPM, SPM, and APM. Further, review articles, letter/personal communication, editorials, case report studies, studies with insufficient data, duplicate studies were excluded.

### 2.3 Data extraction

Two researchers independently reviewed all relevant studies and all parameters. The process of identifying and screening studies for the final datasets was undertaken based on the PRISMA guideline [2].
